# Supplementary material for: Diacylglycerol metabolism drives host-pathogen responses during enteric infection in Drosophila
Source: bioRxiv. 2025 Sep 2:2025.08.28.672769. Preprint. [Version 1] doi: 10.1101/2025.08.28.672769 (PMC12424769; doi:10.1101/2025.08.28.672769)
Supplement: Supplement 1 [file media-1.pdf]

Supplemental information

Diacylglycerol metabolism drives host-pathogen responses during enteric infection in *Drosophila*

Xiaotong Li[1,2*], Mohamed Mlih[2], Jason Karpac[1,2*]

[1]Department of Biology, Texas A&M University, College Station, TX, 77843, USA
[2]Department of Cell Biology and Genetics, College of Medicine, Texas A&M University, Bryan, TX, 77807, USA
*Correspondence: karpac@tamu.edu; lixiaotong@tamu.edu

Table S1: qRT-PCR Primers

Primer	Sequence (5’-3’)
actin5C-F	CTCGCCACTTGCGTTTACAGT
actin5C-R	TCCATATCGTCCCAGTTGGTC
Fasn1-F	AGCTAATAACGGCAGTCAACGG
Fasn1-R	CAGGTTTAGTTGTAGGGGCTAGA
Lipin-F	GTCCGGTACGAAGAAGTCCG
Lipin-R	TCTGAGATACGGCAACTGCT
Mdy-F	ACACAAAGTTCCCAGAGTTCA
Mdy-R	TGGTGATGGTCTCGATTGGA
Drs-F	AAGTACTTGTTCGCCCTCTTCGCT
Drs-R	TCCTTCGCACCAGCACTTCAGACT
Dro-F	TTCACCATCGTTTTCCTGCT
Dro-R	GGCAGCTTGAGTCAGGTGAT
CecA-F	CATTCTGGCCATCACCATTGGACA
CecA-R	ACATTGGCGGCTTGTTGAGCGATT
Def-F	GAGCCACATGCGACCTACTC
Def-R	CAGTAGCCGCCTTTGAACC
AttA-F	TCGTTTGGATCTGACCAAGGGCAT
AttA-R	TTCCGCTGGAACTCGAAACCATTG

Supplementary Figures

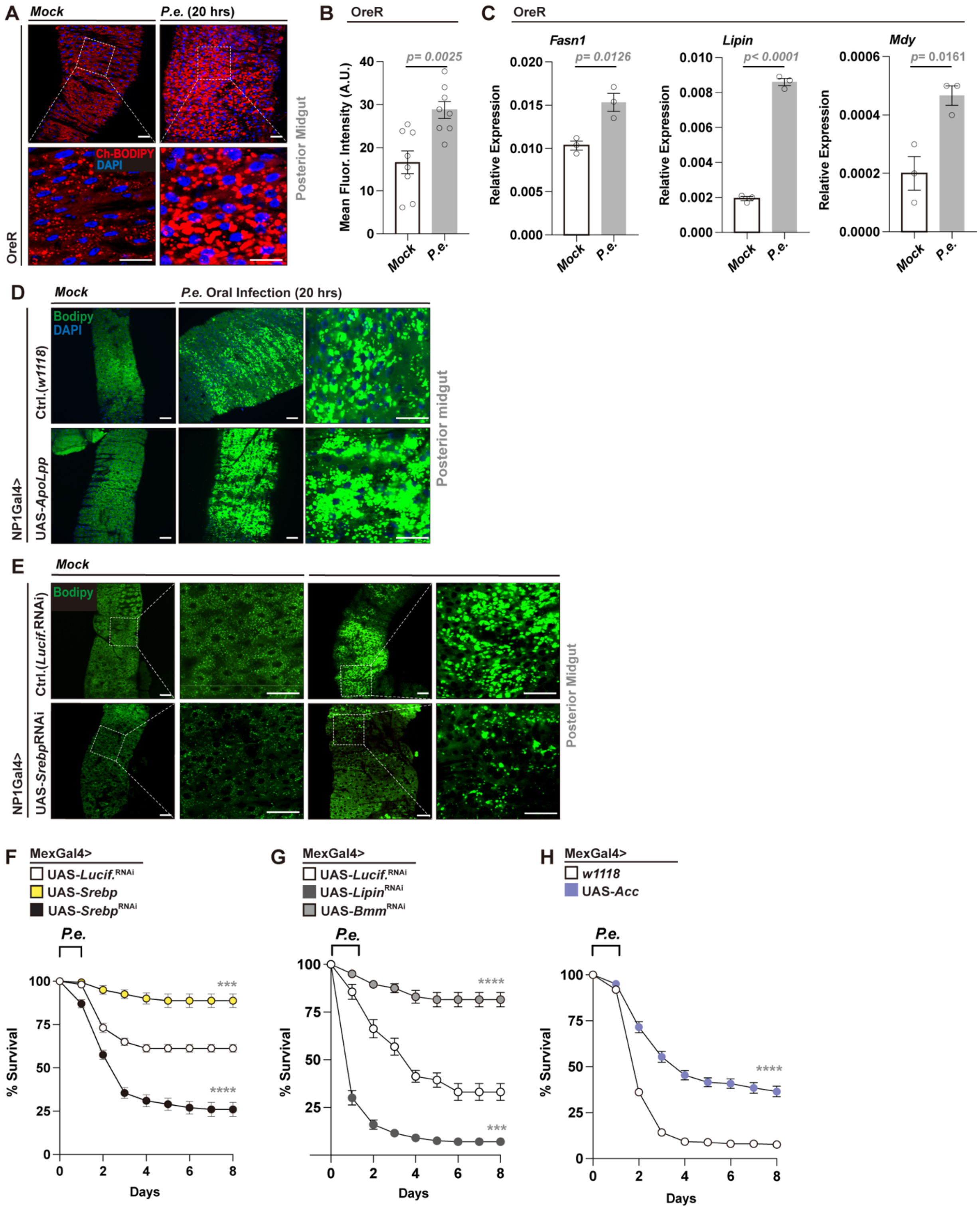

Figure S1. *P.e.* infection alters lipid metabolism in the Drosophila midgut, and enterocyte lipid homeostasis influences host survival post *P.e.* infection. (A, B)

CholEsteryl BODIPY (Ch-BODIPY, red) staining (A) and quantification (B) of cholesterol levels in the posterior midgut of OreR flies during mock or P.e. infection (n = 8 midguts). Nuclei (DAPI, blue). (C) qRT-PCR validation showing *P.e.* infection-induced upregulation of genes involved in lipid anabolism. (D) Enhanced lipid accumulation in NP1Gal4>UAS-*ApoLpp* posterior midgut following infection, revealed by BODIPY 493/503 staining (green). (E) Suppressed lipid accumulation in NP1Gal4>UAS-*SREBP*^{RNAi} flies during infection, as indicated by BODIPY 493/503 staining (green). (F–H) Survival analysis of flies with EC-specific genetic manipulation of lipid metabolism using NP1Gal4 or MexGal4 drivers. Attenuation or overexpression of *Srebp*, *Bmm*, *Lipin*, or *Acc* significantly alters survival compared to NP1Gal4/MexGal4>UAS-*Lucif.*^{RNAi} controls (n = 200 flies/genotype). Statistical comparisons were performed using two-sided t-tests (B, C) or two-way ANOVA (F-H). Bars represent mean ± SEM. ***p value < 0.001, ****p value < 0.0001. Scale bars, 10 µm.

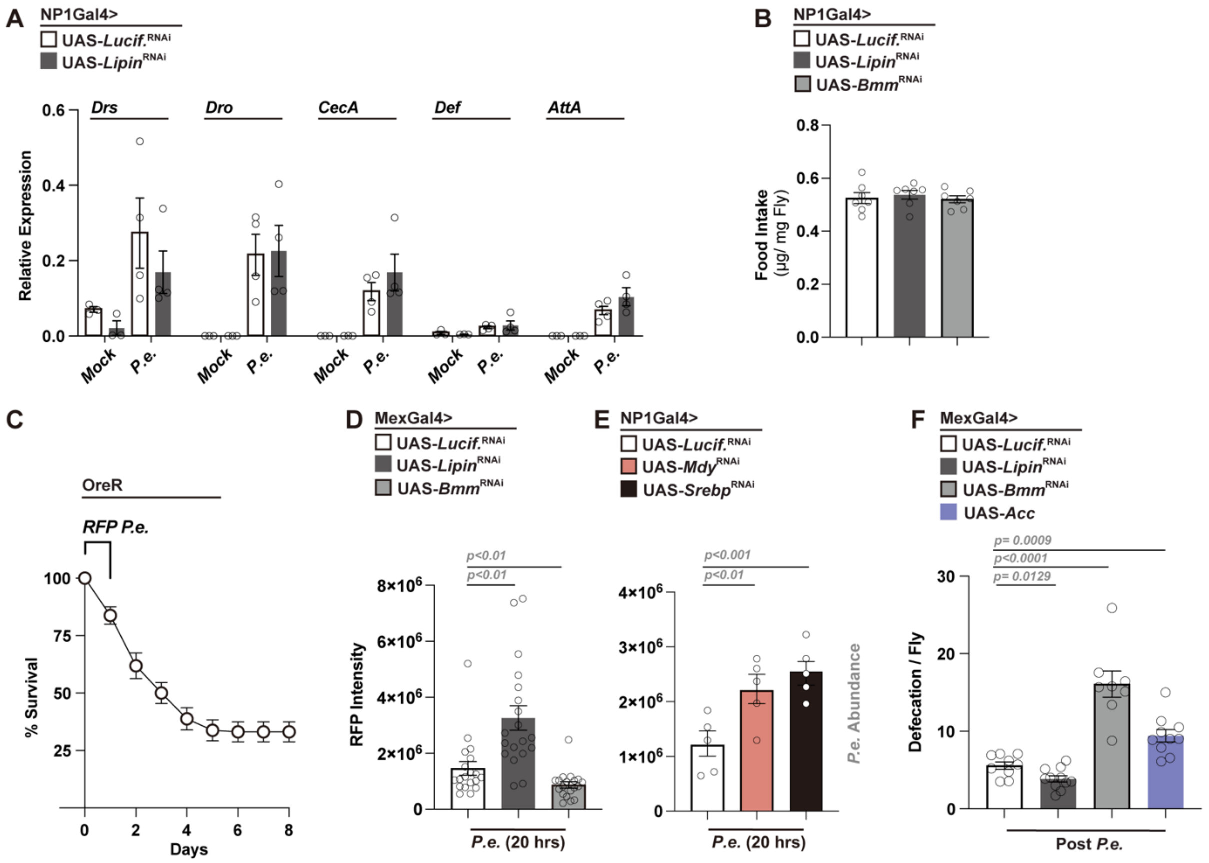

Figure S2. Enterocyte lipid metabolism regulates host survival by modulating gut motility and bacterial clearance. (A) Expression of antimicrobial peptide genes (*Drs, Dro, CecA, Def, Atta*) in midguts of control and *Lipin*^{RNAi} flies shows no significant difference post *P.e.* infection. (B) Food intake remains unchanged in flies with EC-specific attenuation of *Lipin* or *Bmm*. (C) DsRed-expressing *P.e.* (RFP-*P.e.*) is pathogenic and influences host survival after infection. (D, E) EC-specific attenuation of lipid metabolic genes affects bacterial clearance during RFP-*P.e.* infection. (F) Defecation frequency is altered in flies with EC-specific modulation of lipid metabolism. Data represent mean ± SEM. Statistical comparisons were performed using two-sided *t*-tests (A, B, D-F).

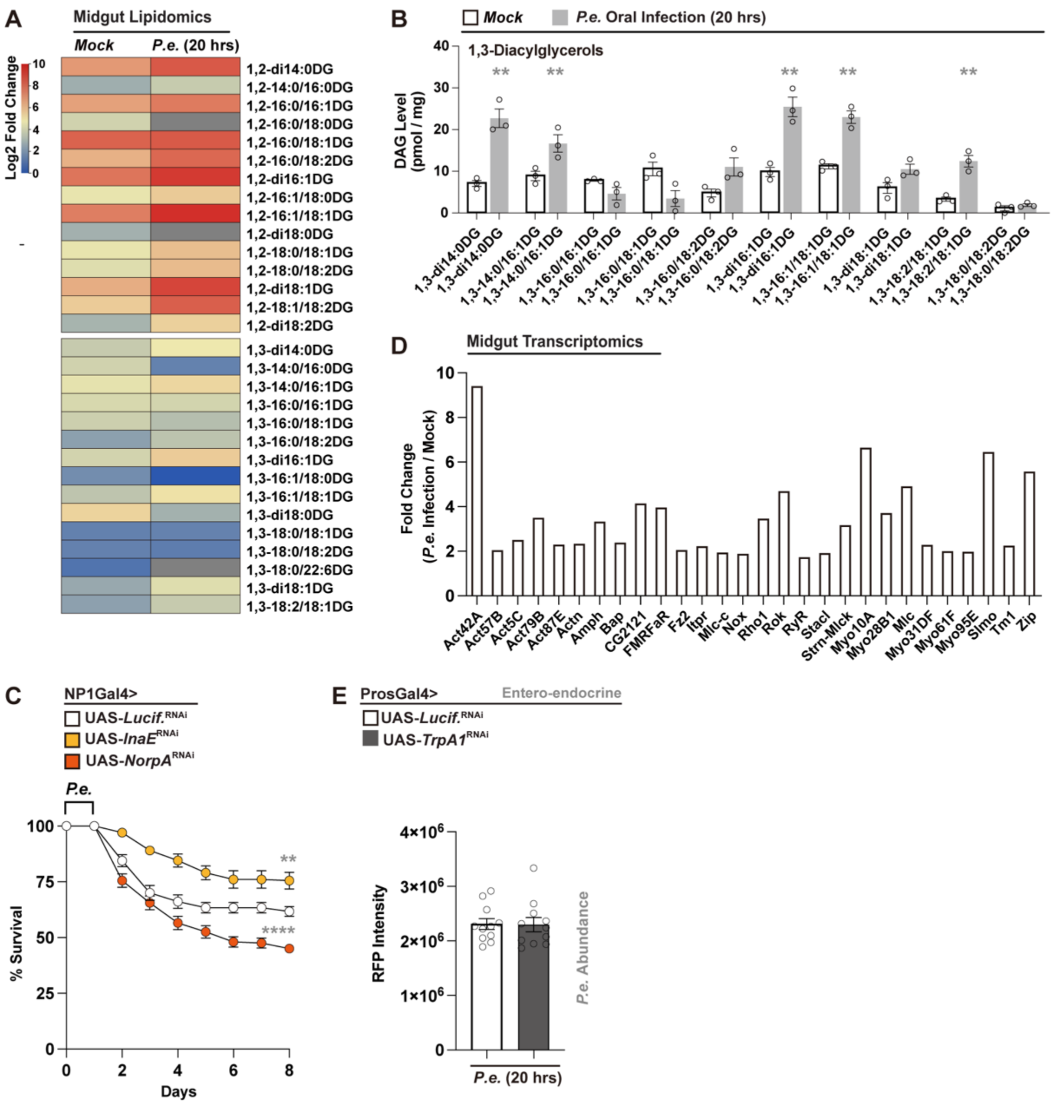

Figure S3. *P.e.* infection induces diacylglycerol (DAG) accumulation and activates visceral muscle remodeling. (A, B) Heatmap (A) and quantification (B) of midgut lipidomic profiles reveal specific elevation of DAGs post *P.e.* infection. Heatmap representation of lipid species in dissected midguts from control (Mock) and *P. e.*–infected flies (20 h post-infection). Genotype: NP1Gal4>UAS-Lucif.^{RNAi}. (C) EC-specific attenuation of *InaE* or *NorpA* alters survival following infection. (D) Gut RNA-seq analysis identifies infection-induced upregulation of muscle contractility-related genes. (E) EEC-specific attenuation of *TrpA1* does not affect bacterial clearance post *P.e.* infection. Statistical comparisons were performed using two-sided t-tests (B, E) or two-way ANOVA (C). Bars represent mean ± SEM. **p value < 0.01, ****p value < 0.0001.

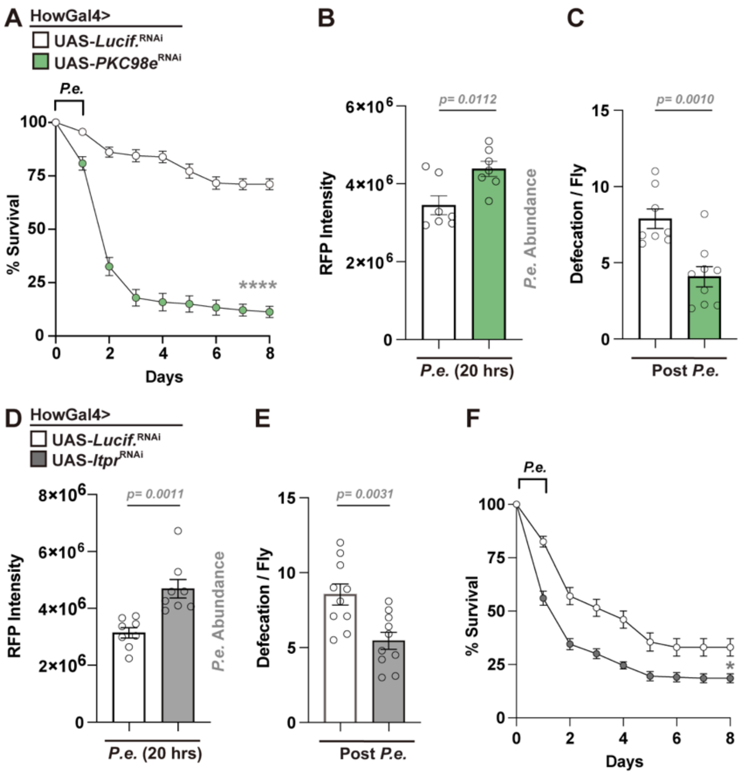

Figure S4. Visceral muscle PKC–calcium signaling regulates muscle contraction, pathogen clearance, and host survival. (A–C) Attenuation of PKC98e in visceral muscle (HowGal4>UAS-*PKC98e*^{RNAi}) reduces survival (A), impairs bacterial clearance (B), and decreases defecation (C) post-infection. (D–F) Visceral muscle-specific attenuation of Itpr (HowGal4>UAS-*Itpr*^{RNAi}) similarly compromises bacterial clearance (D), reduces defecation (E), and lowers survival (F). Statistical comparisons were performed using two-sided *t*-tests (B-E) or two-way ANOVA (A, F). Bars represent mean ± SEM. *p value < 0.05, ****p value < 0.0001.

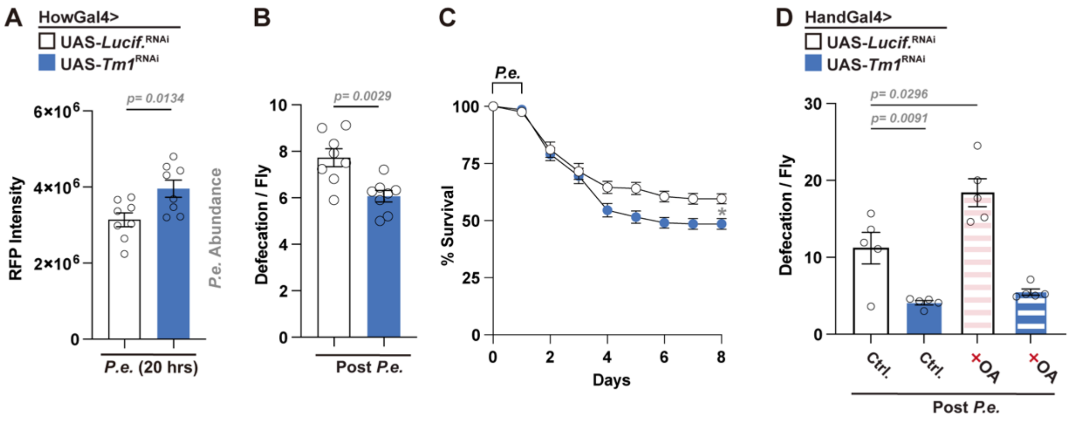

Figure S5. Tropomyosin1 (Tm1) is required in visceral muscle for contraction, pathogen clearance, and host survival. (A, B) Attenuation of Tm1 in visceral muscle (HowGal4>UAS-*Tm1*^{RNAi}) increases midgut bacterial load (A) and reduces defecation frequency (B) post *P.e.* infection. (C) Survival curves showing decreased survival in *Tm1*-deficient flies compared to controls. (D) Dietary OA supplementation prior to infection partially restores defecation in HandGal4>UAS-*Tm1*^{RNAi} flies. Statistical significance determined using two-tailed t-tests (A, B, D) or two-way ANOVA (C). Bars represent mean ± SEM. *p value < 0.05.
